# Epididymal cysts in children: frequency, clinical characteristics, and management strategies

**DOI:** 10.3389/fped.2024.1455866

**Published:** 2024-07-23

**Authors:** Wei Cai, Chenchen Liu, Lei Xu, Qingtao Wu, Tongshuai Kuang, Xiaokun Lin

**Affiliations:** ^1^ Department of Pediatric Surgery, The Second Affiliated Hospital and Yuying Children’s Hospital of Wenzhou Medical University, Wenzhou, Zhejiang, China; ^2^ Department of Pediatric Cardiovascular Medicine, The Second Affiliated Hospital and Yuying Children’s Hospital of Wenzhou Medical University, Wenzhou, Zhejiang, China; ^3^ Wenzhou Key Laboratory of Children Genitourinary Diseases, The Second Affiliated Hospital and Yuying Children’s Hospital of Wenzhou Medical University, Wenzhou, Zhejiang, China

**Keywords:** epididymal cyst, scrotal emergency, children, scrotal ultrasound, conservative management

## Abstract

**Background:**

Epididymal cysts (ECs) are uncommon in the pediatric population. The objective of this study was to evaluate the frequency, clinical characteristics, and management strategies of ECs in children.

**Methods:**

We performed a retrospective review of pediatric scrotal ultrasounds between January 2014 and August 2022 to identify children with ECs.

**Results:**

One hundred and forty-three children boys were found to have ECs, with 95 being pre-pubertal and 48 post-pubertal. The age of the patients ranged from 1 day to 18 years, with a mean age of 10.64 ± 4.55 years. The size of the cysts varied from 2 mm to 35 mm. The most common comorbidities observed were hydrocele, testicular microlithiasis and varicocele. The majority of ECs were detected through routine physical examination. Conservative management was employed for all patients, except for one who required surgical excision. Resolution of ECs occurred in 12 patients, while a reduction in cyst size was observed in 6 cases. Conversely, 2 patients experienced an increase in cyst size, and 6 patients exhibited an increase in cyst number during the follow-up period.

**Conclusion:**

Conservative management is the preferred approach for the majority of cases, with surgical intervention reserved for specific instances.

## 1 Introduction

Epididymal cysts (ECs) are characterized by the accumulation of fluid in either a single sac or multiple sacs, resulting from the dilation of efferent epididymal tubules caused by tubular obstruction (1). The exact cause of ECs remains uncertain. ECs are relatively common in adults but rare in children (2). The first description of ECs in children appears from a 1976 case study (3). However, it has often been observed as a concurrent defect in children with conditions such as cryptorchidism, cystic fibrosis, polycystic kidney disease, and von Hippel-Lindau syndrome (4, 5). ECs can develop in any part of the epididymis, including the head (caput), body (corpus), and tail (cauda). These cysts can also occur bilaterally in testes. Furthermore, the true prevalence of ECs in pediatric populations remains uncertain. While ECs are generally considered rare in childhood, they do occur in a significant number of pubescent boys (6). The advancement of ultrasound (US) technology, particularly the widespread use of color Doppler US in children, has led to an increased detection rate of ECs.

Although it is believed that ECs may resolve spontaneously in children, the potential risk factors for their development remain unknown. The potential disparity in incidence and epidemiological features of ECs between the prepubertal and postpubertal boys remain uncertain. Additionally, the influence of ECs on testicular volume in children of varying ages has yet to be elucidated. Currently, there is a lack of high-quality literature on ECs in childhood, especially concerning optimal management strategies. Therefore, this study aims to retrospectively examine pediatric patients with ECs at our institution to enhance understanding of their clinical characteristics and potential management approaches for pediatric surgeons.

## 2 Patients and methods

### 2.1 Patients

We conducted a retrospective analysis of scrotal US reports in children below 18 years of age at our institution from January 2014 to August 2022, in order to identify a cohort of individuals diagnosed with ECs. Two researchers gathered data independently and then combine it to ensure accuracy and prevent potential biases. We examined age, clinical presentation, comorbidities, and clinical outcomes of these patients. Pubertal stage was assessed by a medical examination and children were classified into two categories: pre-pubertal and pubertal according to Tanner (7). A cystic structure without echo was observed on the epididymis to confirm ECs diagnosis. When ECs were present, sonographic measurements were made of the cyst diameter, as well as the length, width, and transverse diameter of the testicles. Meanwhile, we also compared the testicular dimensions with ipsilateral testis of EC and contralateral normal testis.

### 2.2 Statistical methods

Descriptive analysis was performed using tables and graphs to present the baseline characteristics of the patients. Continuous variables were analyzed using t-test test and categorical variables were compared using Chi-square or Fisher's tests. A *P* value equal to or less than 0.05 was considered significant.

## 3 Results

### 3.1 General clinical data

**Figure 1:**
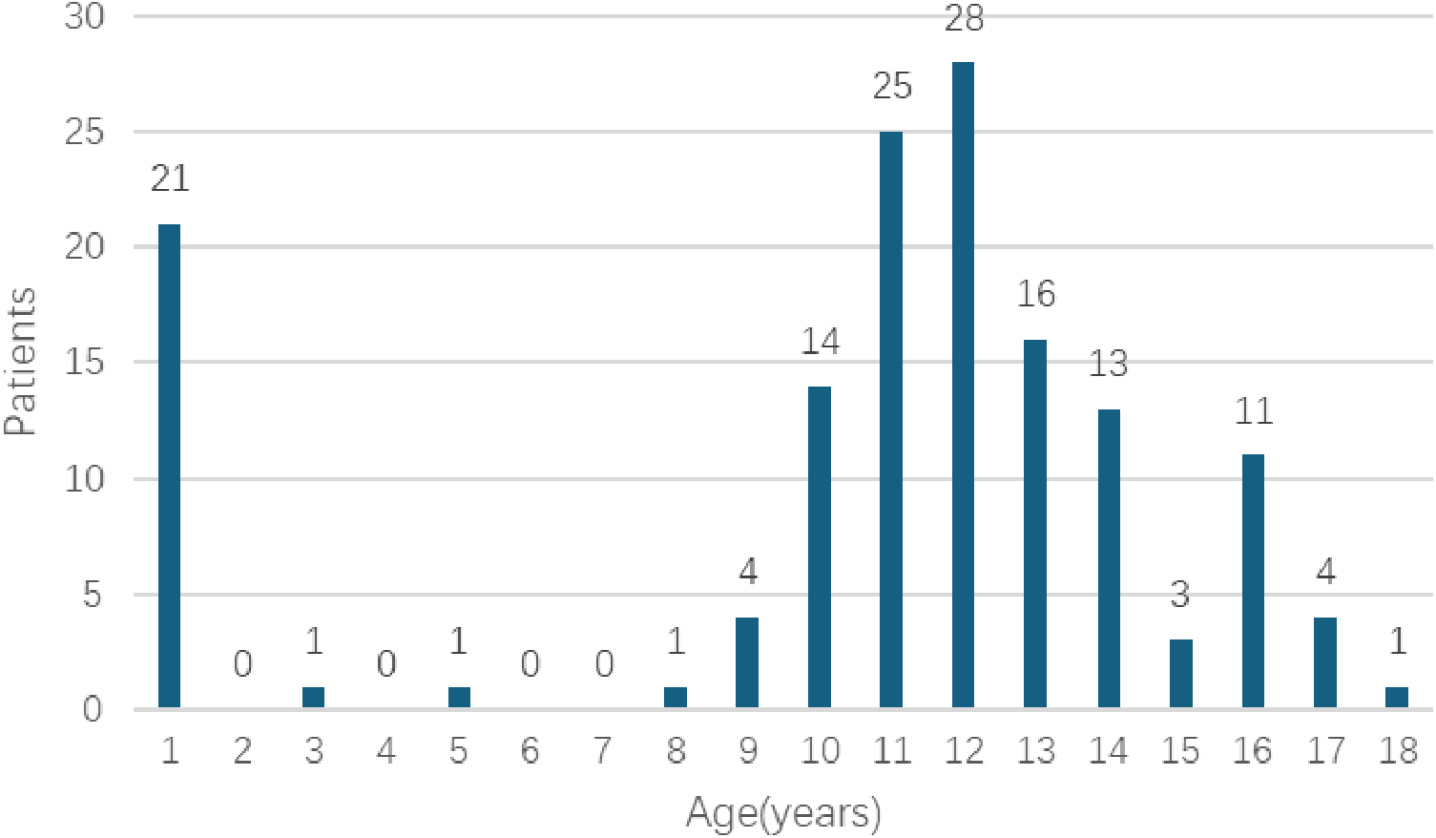
The numbers of patients with each age in 9-year periods from 2014 to 2022.

During the designated study period, scrotal US were conducted on a total of 6,338 patients who were below the age of 18. Among these patients, 143 cases (2.26%) were found to have ECs, as illustrated in Table 1. The age range of the patients varied from 1 day to 18 years, with a mean age of 10.64 ± 4.55 years. It is noteworthy that ECs are more prevalent in children during the pre-pubertal period, with the highest occurrence observed in aged 11–12 years old (Figure 1). 95 patients belonged to the pre-pubertal cohort, while 48 patients were part of the post-pubertal cohort. The average age at presentation was 8.67 ± 4.30 years for the pre-pubertal group and 14.52 ± 1.47 years for the post-pubertal group.

**Table 1:** Comparison of clinical characteristics of epididymal cysts in pre-pubertal vs. pubertal patients.

Characteristic	Pre-pubertal EC	Pubertal EC	*p*
Number	95	48	
Mean age ± SEM (years)	8.67 ± 4.30	14.52 ± 1.47	<0.0001
Location			0.001
Left	44 (46.3%)	15 (31.25%)	
Right	40 (42.1%)	15 (31.25%)	
Bilateral	11 (11.6%)	18 (37.5%)	
Number of cysts			<0.0001
Single	79 (83.2%)	24 (50%)	
Multiple	16 (16.8%)	24 (50%)	
Mean cyst size ± SEM (mm)	4.74 ± 3.22	4.93 ± 2.95	0.741
Region			0.008
Head	89 (93.7%)	41 (85.4%)	
Body	3 (3.2%)	0	
Tail	1 (1.0%)	0	
Multiple	2 (2.1%)	7 (14.6%)	

The distribution of cyst localizations in the pre-pubertal group was as follows: 46.3% (*n* = 44) were located on the left side, 42.1% (*n* = 40) on the right side, and 11.6% (*n* = 11) were bilateral. In the post-pubertal group, 31.25% (*n* = 15) were located on the left side, 31.25% (*n* = 15) on the right side, and 37.5% (*n* = 18) were bilateral. The differences between the two groups were statistically significant. In the pre-pubertal group, a total of 79 (83.2%) single cysts and 16 (16.8%) multiple cysts were observed. Similarly, in the post-pubertal group, there were 24 (50%) single cysts and 24 (50%) multiple cysts. There was a statistically significant difference between groups. The cysts exhibited a range of sizes, spanning from 2 mm to 35 mm, with a mean size of 4.74 ± 3.22 mm in the pre-pubertal group and 4.93 ± 2.95 mm in the post-pubertal group. The head region accounted for the majority of cases (90.9%, *n* = 130), while a small proportion of cases (6.3%, *n* = 9) were found in multiple regions. Additionally, 2.1% (*n* = 3) were located in the body region, and only one case involved the tail region. There was a statistically significant difference between the two groups.

Table 2 offers additional insights into the prevalence of comorbiditles, showing that 53.8% (*n* = 77) of Individuals reported no comorbidities, 41.3% (*n* = 59) reported one comorbidity, and 4.9% (*n* = 7) reported two or more comorbidilies. The most prevalent comorbidities were hydrocele, testicular microlithiasis, and varicocele. Hydrocele was the most common comorbidity in the pre-pubertal group, accounting for 59.1% of cases, followed by testicular microlithiasis at 22.7%. In contrast, varicocele was the most frequent comorbidity in the post-pubertal group, with a proportion of 45.5%. None of these patients exhibited testicular tumors.

**Table 2:** Comparison of comorbidity of epididymal cysts in pre-pubertal vs. pubertal patients.

Comorbidity	Pre-pubertal EC	Pubertal EC	*p*
Number	95	48	1.000
None	51	26	
One	39	20	
Two	5	2	
Type			0.005
Hydrocele	26	5	
Testicular microlithiasis	10	7	
Varicocele	3	10	
Cryptorchidism	2	1	
Inguinal hernia	1	0	
Epididymitis	5	1	
Testicular tumors	0	0	

For those 143 patients presenting with ECs, the primary indication for undergoing scanning was routine physical examination, accounting for 85.3% of cases. The second most common indication was investigations for scrotal mass, including hydrocele (7 cases) and varicocele (4 cases), followed by investigations for cryptorchidism (3 cases). A total of six (4.2%) were performed for scrotal pain, specifically for epididymitis. Pain usually appeared within a few hours to 2 days. Additionally, one patient incidentally discovered an EC during evaluation for blunt trauma to the scrotum.

The relationship between the ECs and testis size is shown in Table 3. There were no statistics difference in testicular length and volume in those with bilateral ECs compared to those with unilateral, or contralateral normal testicle.

**Table 3:** Effect of epididymal cysts on testicular length and volume.

Age	Number	Mean ± SD Length (cm)	*P*	Mean ± SD Vol (cm3)	*p*
0–2 years					
Unilateral EC	19	1.09 ± 0.22	0.707	0.33 ± 0.17	0.652
Contralateral testicle	19	1.12 ± 0.23		0.32 ± 0.15	
Bilateral EC (left)	2	1.05 ± 0.07		0.35 ± 0.04	
Bilateral EC (right)	2	0.95 ± 0.07		0.18 ± 0.06	
3–10 years					
Unilateral EC	19	2.49 ± 0.74	0.625	3.09 ± 2.38	0.181
Contralateral testicle	19	2.49 ± 0.75		2.95 ± 2.52	
Bilateral ECc(left)	2	3.00 ± 0.42		5.91 ± 2.70	
Bilateral EC (right)	2	3.00 ± 0.28		5.96 ± 2.16	
11–14 years					
Unilateral EC	64	3.27 ± 0.60	0.091	6.27 ± 2.77	0.591
Contralateral testicle	64	3.29 ± 0.62		6.19 ± 2.76	
Bilateral EC (left)	18	3.51 ± 0.39		6.87 ± 2.20	
Bilateral EC (right)	18	3.59 ± 0.36		6.97 ± 2.05	
>14 years					
Unilateral EC	12	3.86 ± 0.30	0.396	11.13 ± 2.28	0.847
Contralateral testicle	12	3.87 ± 0.32		10.84 ± 3.16	
Bilateral EC (left)	7	4.09 ± 0.45		11.58 ± 2.31	
Bilateral EC (right)	7	4.03 ± 0.31		11.83 ± 1.99	

### 3.2 Prognosis and follow-up

All patients were managed with conservatively, except for one who underwent surgical excision due to a progressively enlarging mass. Of the patients, follow up is available in 101 and ranges from 6 to 24 months (mean 14.2 months). 41 patients were lost to follow up. Throughout the follow-up period, there was no noteworthy increase in cyst size or number among the 75 children. Complete involution of cysts was observed in 12 children (11.9%), while a decrease in cyst size was found in 6 cases (6%). However, 2 patients (2%) experienced an increase in cyst size, and 6 patients (6%) had an increase in cyst number during the follow-up period, despite being asymptomatic. Notably, no cyst recurrence were observed in patient who underwent surgical excision during the follow-up period.

## 4 Discussion

The true incidence of ECs in children is not currently known. The incidence of ECs has been estimated to range from 5 to 20% (4). Previous studies have indicated that ECs were found in approximately 5% of pediatric patients undergoing scrotal US and in 15% of boys who underwent US due to a palpable mass (8, 9). In our own study, the incidence of ECs in children was approximately 2.26%, which is lower than the rates reported in previous research. Our findings also demonstrated a rise in the prevalence of ECs as individuals age, particularly among aged 11–12 years old, which differed from the previous study (10). However, the underlying cause of ECs during childhood remains uncertain. It is unclear whether these cysts are degenerative in nature or if they arise from the influence of endocrine-disrupting agents during fetal development or postnatally. Additionally, the functional significance of ECs remains unclear. Therefore, a more comprehensive understanding of the etiology and pathogenesis of ECs is necessary to inform clinical management and therapeutic approaches for this condition.

The majority of children with ECs don't exhibit any symptoms. In cases where the cysts are small, they may go unnoticed and are present in 70%–80% of asymptomatic boys (11). However, as ECs increase in size, scrotal swelling becomes the most common clinical manifestation in affected patients. Additionally, some individuals may present with a painless mass on top of the testicle which could be incidentally discovered during a physical examination. Furthermore, It's important to note that certain patients may also exhibit symptoms of testicular pain or orchalgia. These symptoms may be accompanied by the presence of comorbidities. In rare instances, the occurrence of an EC has been observed as a potential cause of acute scrotum, which can mimic testicular torsion (12, 13). In our study, the majority of patients exhibited an absence of symptoms, with only a limited number of individuals presenting with symptoms. Our investigation revealed findings suggesting no indirect association between the symptoms experienced and the size and location of the cysts. Furthermore, our subsequent analysis indicated that ECs may not cause significant changes in testicular size, contradicting previous research findings. It is hypothesized that the presence of ECs and an increase in testicular size in affected patients may be attributed to the hyperplastic effects of endocrine disruptors acting during fetal development (9).

The utilization of US examination is of significant importance in the diagnostic process of ECs (14, 15). This imaging technique not only aids in the identification of the cyst's location within the epididymis, but also facilitates the differentiation between ECs and cystic tumors such as adenomatoid tumor of the epididymis, epidermoid cyst, teratoma of the testis. However, sometimes, it is not easy to make definitive diagnosis with US. Mete reported a case of paratesticular rhabdomyosarcoma (RMS) presenting as an epididymal cyst (16). If uncertainty exists regarding the diagnosis, particularly in cases where the presence of malignant tumors cannot be definitively excluded, it is advisable to conduct enhanced computed tomography (CT) or magnetic resonance imaging (MRI). Furthermore, other potential differential diagnoses encompass epididymitis, orchitis, hydroceles, varicoceles, complicated testicular appendix or epididymal appendices, and spermatocele.

**Figure 2:**
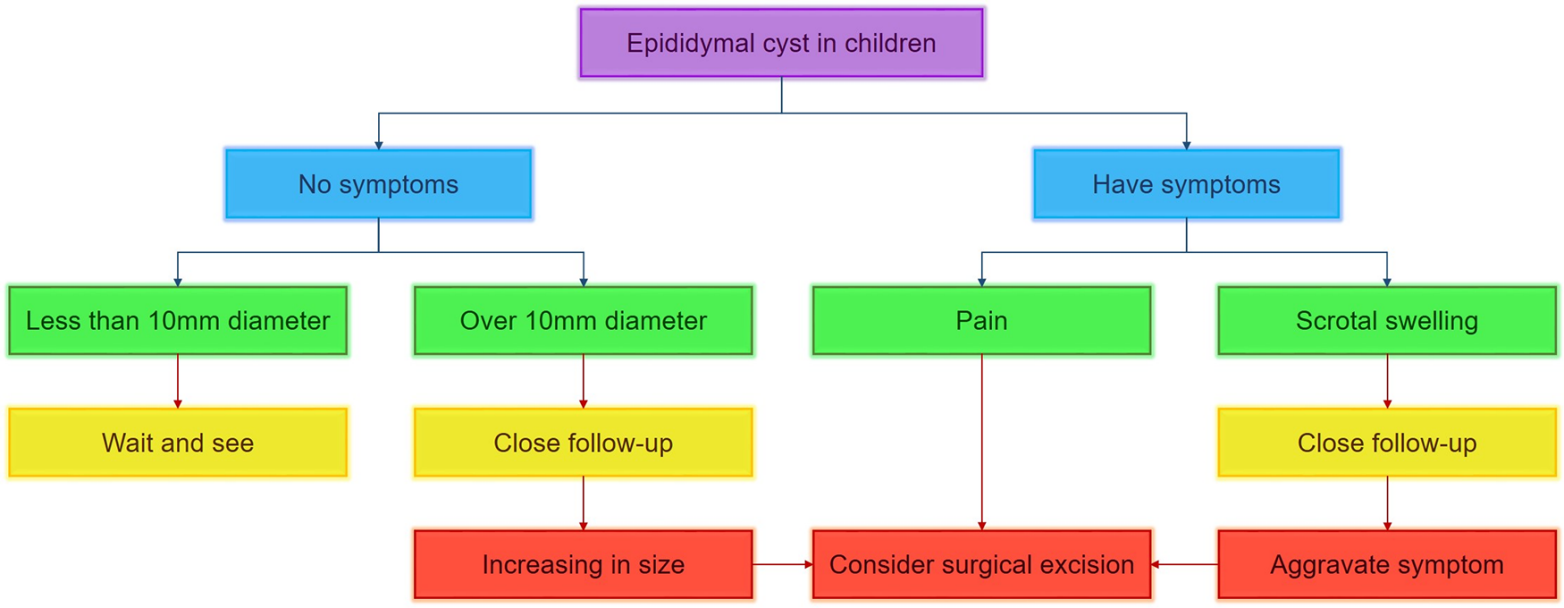
Diagnostic and therapeutic flow chart for management of epididymal cyst in pediatric patients.

The management of ECs encompasses conservative approaches, cyst aspiration, sclerotherapy utilizing various agents such as tetracycline, phenol, sodium tetradecyl sulfate, polidocanol, or ethanolamine oleate, as well as surgical excision (17–22). The choice of treatment for ECs is contingent upon the clinical presentation, cyst size, and age of onset. However, there remains a lack of consensus regarding the optimal therapeutic approach for pediatric patients with ECs. While cyst aspiration and sclerotherapy have been recommended for adult individuals, their efficacy and safety in the pediatric population have yet to be thoroughly investigated (23). Conservative management is typically advised for the majority of pediatric cases due to the benign characteristics and natural regression of these lesions. Specifically, conservative treatment is the preferred approach for an EC measuring less than 10 mm. The duration for complete involution ranges from 4 to 50 months on average (8). Cysts larger than 10 mm in diameter without associated symptoms are generally managed conservatively. Spontaneous regression of epididymal cysts below 3 cm has been documented (24). If an EC exhibits symptoms or exhibits growth, particularly reaching a diameter of 4 cm, surgical removal should be considered (25). For children with ECs whose nature is difficult to determine, timely surgical resection is also necessary to obtain pathology. Furthermore, in specific cases, surgery should be conducted with the aid of microscopic vision to prevent harm to the delicate collecting tubules. Figure 2 presents a diagnostic and therapeutic flow chart for the appropriate management of ECs in pediatric patients.

In this report, we retrospectively analyzed all pediatric patients in order to gain a better understanding of how these children are managed. In general, the prognosis of ECs is favorable. The presence of cysts, regardless of their number or size, did not have a significant impact on patient prognosis. No patient underwent surgery during follow-up nor was admitted to a clinic for acute scrotal pain. However, this study has certain limitations that should be acknowledged. One such limitation is the absence of a true normal control group, which can be considered a restricting factor. Additionally, the retrospective design and relatively short follow-up period for children with ECs, compared to existing literature, may weaken the robustness of this study. To gain more comprehensive insights, future prospective studies are recommended to include a larger sample size and longer follow-up periods. Also, multi-center clinical studies can be established to obtain more reliable data. These studies could potentially yield essential information for devising optimal management strategies for children with ECs.

In conclusion, ECs are relatively uncommon among pediatric patients, typically peaking in incidence around ages 11–12. Most children with ECs are asymptomatic, emphasizing the importance of vigilant monitoring. Conservative management is generally preferred, reserving surgical intervention for symptomatic or enlarging cysts. Future research should focus on larger prospective studies to enhance our understanding and management strategies for this condition in children.

## Data Availability

The original contributions presented in the study are included in the article/Supplementary Material, further inquiries can be directed to the corresponding author.
